# Impact of vancomycin area under the curve in early or later phase on efficacy and nephrotoxicity in patients with enterococcal bloodstream infections: a multicenter study

**DOI:** 10.1186/s12879-024-10399-9

**Published:** 2025-01-28

**Authors:** Piyawadee Tangvichitrerk, Dhitiwat Changpradub, Jatapat Hemapanpairoa, Piraporn Juntanawiwat, Wichai Santimaleeworagun

**Affiliations:** 1 The College of Pharmacotherapy of Thailand, Nonthaburi, 11000 Thailand; 2 Somdetphraphutthaloetla Hospital, Samutsongkharm, 75000 Thailand; 3 Division of Infectious Disease, Department of Medicine, Phramongkutklao Hospital, Bangkok, 10400 Thailand; 4 Department of Pharmaceutical Care, Faculty of Pharmacy, Silpakorn University, Nakhon Pathom, 73000 Thailand; 5 Pharmaceutical Initiative for Resistant Bacteria and Infectious Disease Working Group (PIRBIG), Nakhon Pathom, 73000 Thailand; 6 Department of Clinical Pathology, Division of Microbiology, Phramongkutklao Hospital, Bangkok, 10400 Thailand

**Keywords:** Acute kidney injury, Enterococci, Glycopeptide, PK/PD target, Therapeutic drug monitoring

## Abstract

**Background:**

The optimal pharmacokinetic and pharmacodynamic (PK/PD) parameters of vancomycin that can improve outcomes in enterococcal infections remain controversial. To clarify the therapeutic target for this antibiotic, this study aimed to determine vancomycin PK/PD parameters associated with efficacy in the early (during 72 h) or later (after 72 h) phase of treatment and nephrotoxicity in enterococcal bloodstream infection patients.

**Methods:**

This multicenter retrospective study reviewed medical records of patients with enterococcal bloodstream infections treated with intravenous vancomycin infusion for at least 72 h between January 2016 and March 2024 at Phramongkutklao Hospital or Nopparatrajathanee Hospital in Bangkok, and Rachaburi Hospital in Rachaburi Province, Thailand. Patients with data available on serum vancomycin concentration were analyzed. The primary outcomes were 30-day mortality and acute kidney injury. The estimates of the mean 24-h area under the curve in the first 72 h (AUC_24_) and in steady state (AUC_ss_) were determined by Bayesian theorem.

**Results:**

Overall, 201 vancomycin concentrations were measured within the first 72 h after vancomycin treatment, while 156 were in a steady state (> 72 h). According to Classification and Regression Tree analysis, vancomycin AUC at 420 mg﻿·h/l was the PK/PD target for 30-day mortality. Results reveal that patients with AUC_24_ (early phase) and AUC_ss_ < 420 mg﻿·h/l (later phase) had significantly higher 14-day, 30-day, and in-hospital mortality than AUC ≥ 420 mg﻿·h/l groups. In addition, patients with AUC_24_ ≥ 420 mg﻿·h/l in the early phase had significantly reduced microbiological failure (*p* = 0.004). Patients with AUC ≥ 700 mg﻿·h/l in early and later phases had significantly increased acute kidney injury risk. In addition, patients receiving concomitant nephrotoxic drugs had an AUC cutoff value of 650 mg﻿·h/l. Multivariate Cox regression analysis showed that vancomycin AUC_ss_ < 420 mg﻿·h/l, unknown source of bacteremia, and acute kidney injury were significantly associated with 30-day mortality.

**Conclusions:**

AUC 420–650 mg﻿·h/l in early and later phases was the target of vancomycin’s PK/PD in enterococcal bacteremia patients for efficacy and to prevent acute kidney injury. This study suggests close monitoring of vancomycin levels to ensure efficacy and safety.

## Background

Enterococci are Gram-positive facultative anaerobic bacteria that can cause bloodstream infection in community-associated and hospital-associated clinical settings. These bacteria are many causes of bacteremia, endocarditis, intra-abdominal infection, skin and soft-tissue infection, urinary tract infection, and meningitis [1]. The mortality rate of enterococcal bloodstream infection (BSI) was approximately 11%–48% [2–6]; ampicillin resistance being a significant risk factor that increases such mortality [7]. Notably, the 30-day mortality rate of ampicillin-resistant vancomycin-susceptible enterococci (AR-VSE) in Thailand was reported to be 38.7% [8].

Vancomycin, a glycopeptide antibiotic, remains the treatment of choice for Gram-positive resistant infections, such as those of *Enterococcus* spp., *Staphylococcus* spp., and *Streptococcus* spp. Due to the risk of resistance and increased toxicity, vancomycin should be reserved for AR-VSE infections or patients with a confirmed β-lactam allergy. Treatment with glycopeptides revealed a higher clinical cure rate compared with daptomycin; however, mortality did not differ significantly between the groups [9]. Optimization of the dosing regimen is essential to ensure the efficacy and safety of vancomycin. Moreover, vancomycin pharmacokinetic and pharmacodynamic (PK/PD) targets for treatment of methicillin-resistant *Staphylococcus aureus* (MRSA) infection are well established, with an area under the curve above the minimum inhibitory concentration (AUC/MIC) ratio of 400–600 mg﻿﻿·h/l for efficacy and safety, assuming a vancomycin MIC determined by broth microdilution [BMD] of 1 mg/l [10].

Few studies have reported about optimal PK/PD targets for non-MRSA infections, especially enterococcal infection. A recent study by Jumah et al. evaluated the association between AUC_0–24_/MIC_Etest_ value of ≥ 389.09 mg﻿﻿·h/l and lower 30-day all-cause mortality in 57 patients with enterococcal bacteremia [11]. Meanwhile, Katip et al. showed that vancomycin AUC/MIC of ≥ 400 mg﻿﻿·h/l was associated with improved clinical response and microbiological response in enterococcal infection [12]. Two other studies found no significant correlation between AUC/MIC and mortality; however, an association was identified between lower trough concentration (Ctr) and higher mortality [13, 14]. The findings showed that the achieved PK/PD target of vancomycin was not a significant risk factor for mortality. It also revealed that acute kidney injury, corticosteroid treatment, and unknown source of bacteremia were associated with increased mortality in enterococcal bloodstream infection [15].

Besides related to treatment efficacy, AUC was also associated with nephrotoxicity. Vancomycin AUC/MIC of ≥ 400 mg﻿﻿·h/l was shown to be associated with a higher risk of acute kidney injury (AKI) than vancomycin AUC/MIC of < 400 mg﻿﻿·h/l (14.96% vs. 3.85%) in patients with enterococcal infection [12]. Moreover, the incidence of vancomycin-associated AKI was correlated with exposure at a steady state in MRSA infection [16, 17].

Few studies have examined vancomycin PK/PD targets in enterococcal infection to improve outcomes. Thus, this study aimed to determine vancomycin PK/PD parameters associated with efficacy in either early (during 72 h) or later (after 72 h) phase and with nephrotoxicity in patients with enterococcal bloodstream infection.

## Methods

### Study design

This multicenter retrospective study was conducted between January 2016 and March 2024 at Phramongkutklao Hospital and Nopparatrajathanee Hospital in Bangkok, and Rachaburi Hospital in Rachaburi Province, Thailand. The study included patients admitted with AR-VSE bloodstream infection and treated with intravenous vancomycin infusion for at least 72 h (for patients with more than one episode of enterococcal infection, only the first episode was included), for whom data on serum vancomycin concentration were available. The exclusion criteria were patients younger than 20 years, pregnant, who received combination therapy or anti-ampicillin-resistant *Enterococcus* activity, and incomplete patient data.

### Ethics approval, data collection, and outcomes

This study was approved by the Institutional Review Board of the Royal Thai Army Medical Department at Phramongkutklao College of Medicine and Phramongkutklao Hospital, Human Research Ethics Committee of Ratchaburi Hospital, and Human Research Ethics Committee of Nopparatrajathanee Hospital. Data were collected after ethical approval and with permission from the hospital director.

The patient data obtained from the electronic medical records included: 1) demographic data, such as sex, age, body weight, underlying disease, and Charlson Comorbidity Index; 2) infection data, such as infection site, *enterococcus* species, susceptibility result, coinfecting organisms, antibiotic treatment for AR-VSE bacteremia, and source control; 3) severity of illness, admitting ward, septic shock, critically ill status, mechanical ventilation use, Sequential Organ Failure Assessment score, and Acute Physiology and Chronic Health Evaluation II score at the date of receiving vancomycin; 4) clinical outcomes, including 14- and 30-day mortality, in-hospital mortality, and clinical failure including patients who received add-on therapy for enterococcal bloodstream infection or who had clinical signs and symptoms of worsening *Enterococcus* spp. infection after at least 72 h of antimicrobial therapy; 5) microbiological failure such as *Enterococcus*-positive blood culture after 72 h of antimicrobial therapy; and 6) AKI (increase in serum creatinine ≥ 0.3 mg/dl from baseline within 48 h or increase ≥ 1.5 times baseline within the prior 7 days or urine volume < 0.5 ml/kg/h for 6 h) [18].

Patients received loading doses with 20–35 mg/kg per dose, according to actual body weight. The maintenance dose was 15–20 mg/kg every 12 h in patients with normal renal function. For patients with renal impairment, the vancomycin dosing interval was adjusted based on creatinine clearance (CrCl): patients with CrCl ≥ 50 ml/min, 15–49 ml/min, and < 15 ml/min were dosed at intervals of 12, 24, 48, respectively. For patients with hemodialysis (HD), the vancomycin interval was 72 h (or after HD) [10]. The vancomycin concentration were monitored after the initial dose and after adjusted the dosing regimen by clinical pharmacist. There were two methods for measuring vancomycin concentration: 1) collection of two-point (peak-trough) concentration at 2 h after infusion and ≤ 30 min before the next dose; 2) collection of one-point concentration (trough). The administration of vancomycin dosing regimens and therapeutic drug monitoring were recorded in PrecisePK software (San Diego, CA, USA) by clinical pharmacists. The dates and times of vancomycin administration and the vancomycin concentrations during blood sampling were recorded and submitted to the PrecisePK software. The AUC of vancomycin in the first 72 h (AUC_0-72_) was calculated from PrecisePK software version 2.4.0 based on Bayesian algorithms to achieve the target in the early phase of infection, especially in bacteremia [19]. The mean daily AUC_0-72_ determined AUC_24_. The vancomycin AUC at a steady state (> 72 h, later phase) was determined at five half-lives or the last day of vancomycin administration if stopped earlier.

Trough concentration within 72 h (Ctr_72h_) was determined by the mean of the vancomycin trough within 72 h for individual patients, while trough concentration at a steady state (Ctr_ss_) was determined by the mean trough concentration at > 72 h.

### Microbiological data

The MIC of antimicrobial agents was determined using automated susceptibility testing (Sensititre™ Aris HiQ System) based on the broth microdilution method reported by the hospital’s laboratory. Clinical and Laboratory Standards Institute M100-ED33 2023 was used to classify sensitivity to ampicillin or vancomycin [20].

### Vancomycin assay

Serum vancomycin concentrations were determined using a fluorescence immunoassay (VANC3, Cobas; Roche Diagnostics, Indianapolis, IN, USA) for Phramongkutklao Hospital and Ratchaburi Hospital. Whereas at Nopparatrajathanee Hospital, Particle-enhanced turbidimetric inhibitor immunoassay methods (ALINITY C; Abbott, Abbott Park, IL, USA) were used to determine vancomycin levels. The limit of detection of the two assays was 4 mg/l and the coefficient of variation was ≤ 10%.

### Statistical analysis

Descriptive statistics for clinical characteristics such as the MIC 50th percentile (MIC_50_) and 90th percentile (MIC_90_) were used. The AUC cutoff value for vancomycin treatment outcome and nephrotoxicity were analyzed by Classification and Regression Tree (CART). Chi-square test, independent t-test, or Mann–Whitney U test was used for comparisons of categorical and continuous variables between groups. The survival analysis was estimated by Kaplan–Meier curve. Multivariate analyses by logistic regression were used to explore factors associated with mortality. *P* < 0.05 was considered statistically significant as analyzed by Statistical Package for the Social Sciences version 27.0. (IBM TechXchange Community; Armonk, New York, USA).

## Results

Overall, 112 enterococcal bacteremia participants who received vancomycin with 357 vancomycin concentrations between January 2016 and March 2024 were evaluated. Among those, 67 cases (59.82%) were male, the mean age was 63.50 years, and there were 75 (66.96%) critically ill patients. Fifteen cases were reported on hemodialysis, and 3 cases were reported on continuous renal replacement therapy. Diabetes and malignancy were the most common underlying diseases. Of the 112 participants, 53 (47.32%) had an unknown source of bacteremia. Catheter-related (32; 28.57%) and urinary tract infections (13; 11.61%) were the two most common sources of bacteremia (Table 1).

**Table 1:** Baseline characteristics of patients with enterococcal bacteremia treated with vancomycin (*n* = 112 participants)

Characteristics	Values
Age, mean ± SD (years)	63.50 ± 17.56
Male, n (%)	67 (59.82)
Body weight, mean ± SD (kg)	60.53 ± 15.32
Body mass index, mean ± SD (kg/m^2^)	22.96 ± 5.67
Scr, mean ± SD (mg/dL)	1.83 ± 1.64
**Source of bacteremia**	
Catheter-related, n (%)	32 (28.57)
Urinary tract, n (%)	13 (11.61)
Intra-abdominal, n (%)	9 (8.04)
Skin and soft tissue, n (%)	4 (3.57)
Unknown, n (%)	53 (47.32)
**Source control**	40 (35.71)
**Comorbidities**	
Diabetes, n (%)	34 (30.36)
Malignancy, n (%)	40 (35.71)
Cardiovascular disease, n (%)	14 (12.50)
Chronic kidney disease, n (%)	11 (9.82)
Autoimmune disease, n (%)	8 (7.14)
Cerebrovascular disease, n (%)	7 (6.25)
Liver disease, n (%)	6 (5.36)
Charlson comorbidity index, mean ± SD	2.23 ± 2.04
**Severities**	
Critically ill, n (%)	75 (66.96)
APACHE II score, mean ± SD	17.54 ± 6.55
SOFA score, mean ± SD	6.65 ﻿± 5.23
**Vancomycin**	
Loading dose, mean ± SD (mg/kg)	25.42 ± 6.35
Maintenance dose ± SD (mg/kg/day)	
CrCl ≥ 50 ml/min (*n* = 47)	32.64 ± 11.97
CrCl 15—< 50 ml/min (*n* = 40)	20.16 ± 8.85
CrCl < 15 ml/min (*n* = 7)	16.98 ± 10.54
Hemodialysis (*n* = 15)	11.91 ± 7.23
CRRT (*n* = 3)	30.50 ± 6.30
Duration, mean ± SD (day)	9.66 ± 5.87
Ctr_72h_, mean ± SD (mg/l)	14.89 ± 6.98
Ctr_ss_, mean ± SD (mg/l)	22.09 ± 11.23
**Concomitant nephrotoxic drugs**, n (%)	
Colistin	29 (25.89)
Amphotericin B	9 (8.03)
Aminoglycosides	7 (6.25)
Co-trimoxazole	6 (5.36)
Piperacillin/tazobactam	5 (4.46)
***Enterococcus* species**, n (%)	
*Enterococcus faecium*	109 (97.92)
*Enterococcus* spp.^a^	3 (2.68)
**Co-infection organism, n (%)**	**30 (26.79)**
*Enterobacterales*	18 (16.07)
*Acinetobacter baumannii*	5 (4.46)
*Candida* spp.	5 (4.46)
*Pseudomonas aeruginosa*	2 (1.79)
*Pseudomonas* spp.	2 (1.79)
*Stenotrophomonas maltophilia*	1 (0.89)

^a^*Enterococcus* spp.: *E. faecalis*, *E. ruffinosus, E. avium*, *Scr* serum creatinine, *APACHE* acute physiology and chronic health evaluation, *SOFA* sequential organ failure assessment, *SD* standard deviation, *Ctr*_*72h*_ mean trough concentration in the first 72 h, *Ctr*_*ss*_ mean trough concentration in a steady state, *CrCl* creatinine clearance, *CRRT* continuous renal replacement therapy

All *enterococcus* species were resistant to ampicillin and susceptible to vancomycin. A total of 109 (97.92%) of the enterococci were identified as *Enterococcus faecium* (Table 1). One hundred-fifth patients reported MIC, vancomycin had a MIC distribution of 0.25–2 mg/l, with MIC_50_ and MIC_90_ values of 1 mg/l.

Overall, 201 vancomycin concentrations were measured within the first 72 h from 106 participants, while 156 concentrations from 84 participants were obtained in a steady state (> 72 h) after vancomycin treatment. The total mean vancomycin therapy duration was 9.66 days (3–46 days). Mean trough concentrations during the first 72 h and in a steady state were 14.89 mg/l (< 4 to 41 mg/l) and 22.09 mg/l (7 to 63.4 mg/l), respectively. The pharmacokinetic parameters of vancomycin were derived from the PrecisePK program (Table 2).

**Table 2:** Pharmacokinetic parameters of vancomycin from 112 participants^a^

Parameters^b^	Mean	Median	Minimum	Maximum	S.D
Vc (L)	14.76	10.20	6.01	100.29	14.20
Vp (L)	35.70	31.91	12.29	101.58	16.00
Ke_12_ (h^−1^)	1.33	1.33	0.07	4.33	0.73
Ke_21_ (h^−1^)	0.40	0.46	0.11	0.46	0.12
Half-life (h)	32.09	11.87	3.69	192.46	39.61
CL (L/h)	2.96	2.37	0.24	10.47	2.18

^a^Population parameter derived from PrecisePK software, ^b^Vc = volume of the central compartment, Vp = volume of the peripheral compartment, Ke_12_ = elimination rate constant from central to peripheral compartment, Ke_21_ = elimination rate constant from peripheral to central compartment, CL = total clearance of vancomycin

**Fig. 1:**
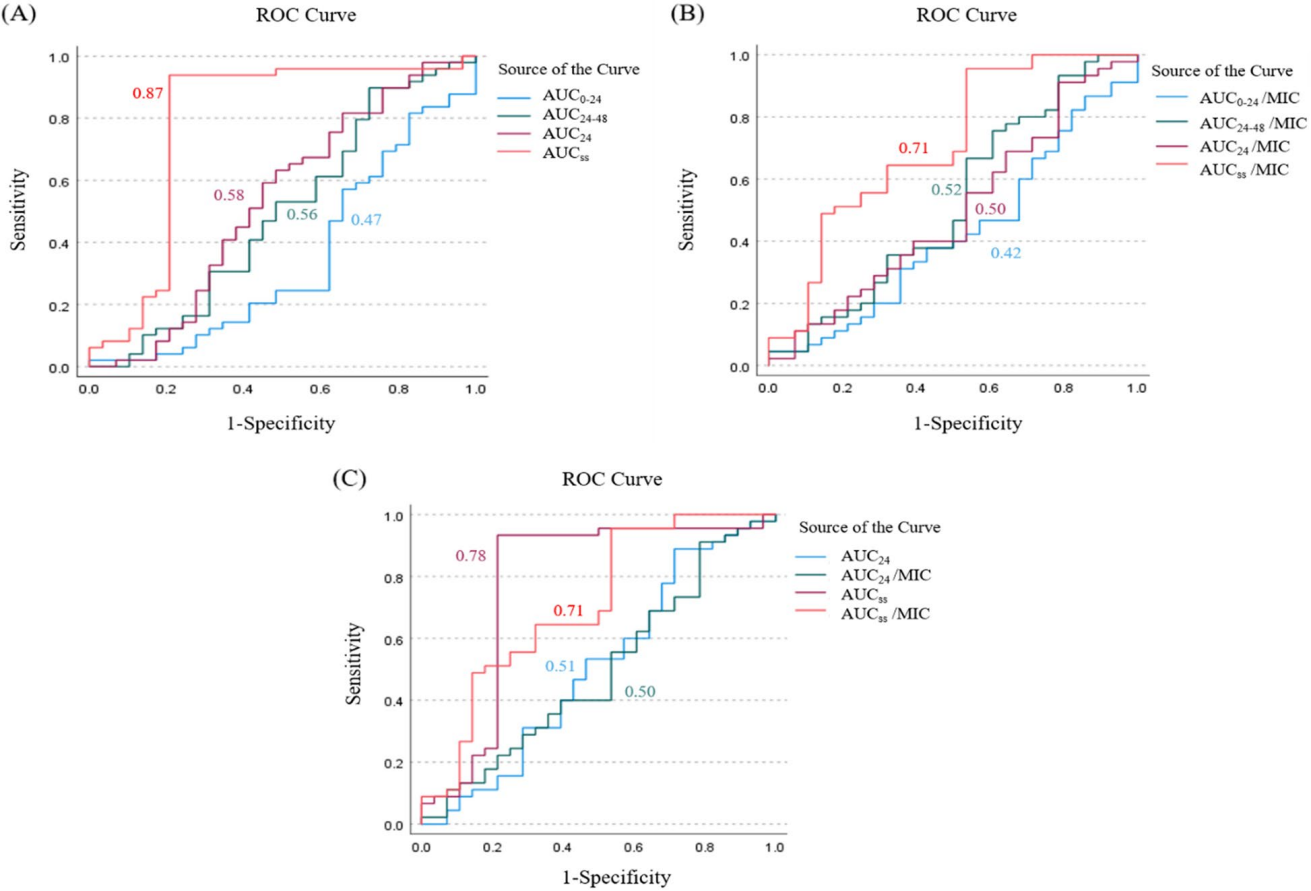
The receiver operating characteristic (ROC) curve of vancomycin AUC for predicting 30-day mortality: The ROC curve of vancomycin AUC (**A**), The ROC curve of vancomycin AUC/MIC (**B**), The ROC curve of vancomycin AUC and AUC/MIC (**C**)

The numbers of cases associated with 14-day, 30-day, and in-hospital mortality were 27 (24.11%), 47 (41.96%), and 62 (55.34%), respectively. Repeated blood cultures were performed for only 87 participants after treatment. The microbiological and clinical failure rates were 1.8% (2 of 87 cases) and 9.8% (11 of 112 cases), respectively. According to CART analysis, the vancomycin AUC breakpoint for 30-day mortality was 420 mg﻿·h/l. The receiver operating characteristic (ROC) curve of AUC at the first 24 h (AUC_0–24_), AUC at 24–48 h (AUC_24–48_), AUC_24_, and AUC in a steady state (AUC_ss_) were 0.47 (OR 0.982, *p* = 0.971), 0.56 (OR 0.318, *p* = 0.012), 0.58 (OR 0.218, *p* = 0.003), and 0.87 (OR 0.014, *p* < 0.001), respectively at the breakpoint. For AUC/MIC, the ROC curve of AUC_ss_/MIC was the highest capacity (0.710), and the breakpoint for 30-day mortality was 412 mg﻿·﻿h/l. To compare AUC and AUC to MIC ratios, AUC had the highest capacity to predict 30-day mortality in the early and later phases (Fig. 1).

For efficacy, the patients with AUC_24_ < 420 mg﻿·h/l had significantly higher 14-day, 30-day, in-hospital mortality, and microbiological failure rates than AUC_24_ ≥ 420 mg﻿·h/l groups during the first 72 h of vancomycin administration, while no significant difference was observed in nephrotoxicity (61.54% vs. 47.23%, respectively; *p* = 0.344) (Table 3). Trough concentration within 72 h (Ctr_72h_) was not correlated with clinical outcomes.

**Table 3:** Outcomes of vancomycin AUC in patients with enterococcal bacteremia

Outcomes n(%)	Vancomycin AUC_24_ (mg·h/L)		*p*-value	Vancomycin AUC_ss_ (mg·h/L)		*p*-value
	**< 420** **(*n* = 20)**	**≥ 420** **(*n* = 86)**		**< 420** **(*n* = 30)**	**≥ 420** **(*n* = 54)**	
14-day mortality	9 (45.00)	15 (17.44)	0.008*	13 (43.33)	4 (7.41)	< 0.001*
30-day mortality	14 (70.00)	29 (33.72)	0.003*	27 (90.00)	6 (11.11)	< 0.001*
Hospital mortality	17 (85.00)	42 (48.84)	0.003*	28 (93.33)	18 (33.33)	< 0.001*
Clinical failure	2 (10.00)	9 (10.47)	0.951	4 (13.33)	3 (5.56)	0.217
Microbiological failure^a^	2/17 (11.76)	0/67 (0.00)	0.004*	1/21 (4.8)	0/45 (0.00)	0.140
Nephrotoxicity^b^	3/6 (50.00)	20/47 (42.55)	0.729	5/14 (35.71)	10/27 (37.04)	0.934

^*^p value < 0.05*, *^a^84 patients had vancomycin concentration at first 72 h, and 66 patients at steady state with repeated blood culture were analyzed for microbiological failure. ^b^53 patients had vancomycin concentration at first 72 h, and 41 patients at steady state with non-renal replacement therapy, serum creatinine following vancomycin administration were reported, and those who did not receive concomitant nephrotoxic drugs were analyzed for nephrotoxicity

**Fig. 2:**
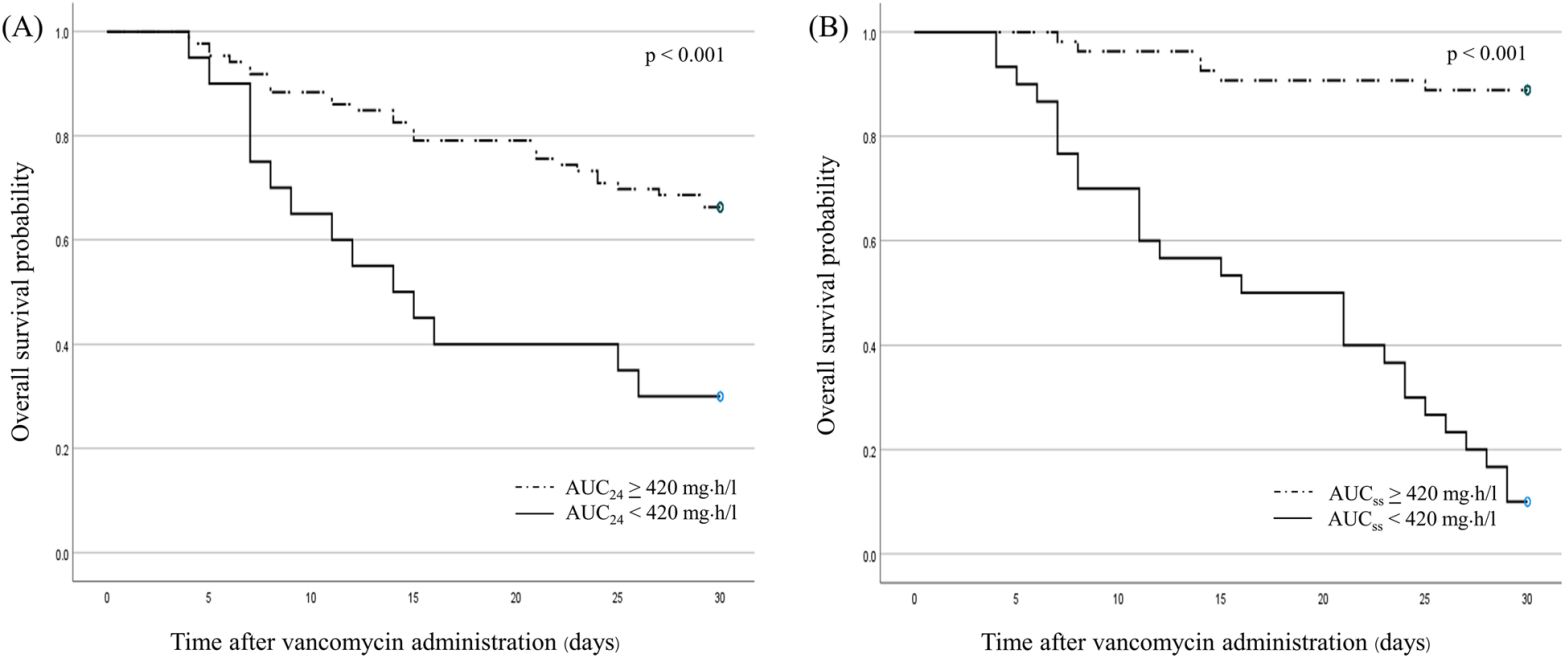
Kaplan–Meier survival curves of patients with vancomycin AUC_24_ < 420 mg﻿·h/l and AUC_24_ ≥ 420 mg﻿·h/l (**A**), AUC_ss_ < 420 mg﻿·h/l and AUC_ss_ ≥ 420 mg﻿·h/l (**B**)

In a steady state of vancomycin, patients with AUC_ss_ < 420 mg·h/l had significantly higher 14-day, 30-day, and in-hospital mortality rates than those with AUC_ss_ ≥ 420 mg﻿·h/l. No significant differences were observed in clinical and microbiological failure rates (Table 3). Trough concentration at a steady state (Ctr_ss_) < 13 mg/l was associated with significantly higher 14-day mortality than that in patients with Ctr_ss_ ≥ 13 mg/l (38.89% vs. 15.15%, *p* = 0.026, respectively). The 30-day all-cause mortality tended to be higher in patients with Ctr_ss_ < 13 mg/l; however, no significant difference was observed (55.56% vs. 34.85%, *p* = 0.111). Kaplan–Meier survival analysis of the time to death demonstrated significantly decreased mortality in the AUC_24_ and AUC_ss_ ≥ 420 mg﻿·h/l groups (AUC_24_: HR 0.34; 95% CI 0.18–0.64, *p* < 0.001, AUC_ss_: HR 0.06; 95% CI 0.03–0.16, *p* < 0.001) (Fig. 2).

**Fig. 3:**
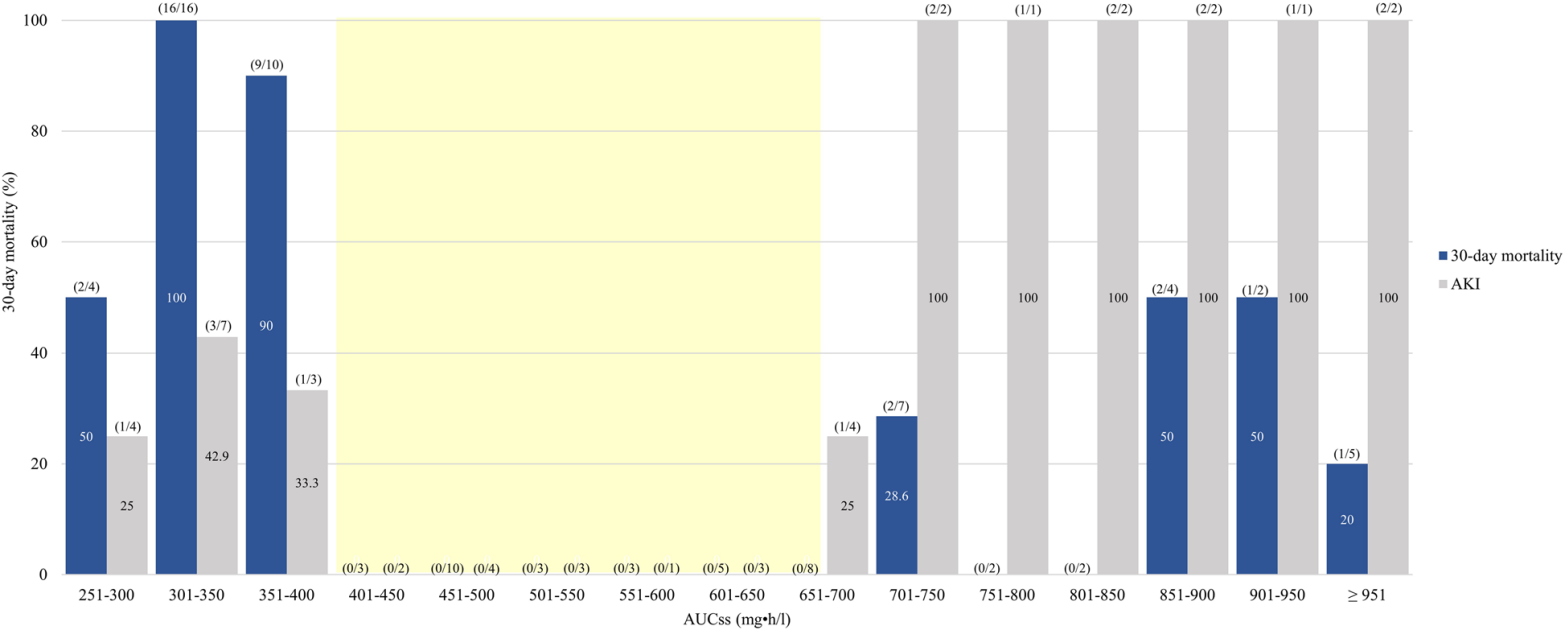
Correlation between 30-day mortality (blue bar), AUC_ss_, and acute kidney injury (gray bar)

Fifty-five patients (50%) had developed AKI during vancomycin treatment. Fifty-six patients who did not receive renal replacement therapy and serum creatinine following vancomycin administration were reported and those who did not receive concomitant nephrotoxic drugs were analyzed for AUC-related nephrotoxicity. Overall, in 25 patients with AKI, according to CART analysis, the vancomycin AUC breakpoint for AKI was 700 mg﻿·h/l (ROC curve 0.826). This breakpoint shows a significantly higher nephrotoxicity rate in the first 72 h (AUC_24_ < 700 mg﻿·h/l: 28.57% versus AUC_24_ ≥ 700 mg﻿·h/l: 72.22%, *p* < 0.002) and steady-state (AUC_ss_ < 700 mg﻿·h/l: 19.35% versus AUC_ss_ ≥ 700 mg﻿·h/l: 90%, *p* < 0.001). In subgroup analysis for patients who receive concomitant nephrotoxic drugs (34 patients), the AUC cutoff value was shipped to 650 mg﻿·h/l (ROC curve 0.689). The correlation of AUC_ss_ and 30-day mortality or AKI indicates that vancomycin has a therapeutic range of 420–650 mg﻿·h/l with high efficacy against enterococcal bloodstream infection and less nephrotoxicity (Fig. 3).

The multivariate Cox regression analysis showed that vancomycin AUC_ss_ < 420 mg·h/l (OR 44.65; 95% CI 5.59–356.84, *p* < 0.001), unknown source of bacteremia (OR 8.28; 95% CI 1.86–36.90, *p* = 0.006), and AKI (OR 5.72; 95% CI 1.32–24.88, *p* = 0.020) were associated with 30-day mortality in patients with enterococcal bloodstream infection (Table 4).

**Table 4:** Univariate and multivariate logistic regression model of factors associated with 30-day mortality

Characteristic	Univariate analysis			Multivariate analysis		
	**OR**	**95%CI**	***p*-value**	**OR**	**95% CI**	***p*-value**
Critically ill	11.09	3.57–34.46	< 0.001*	4.93	0.84–28.85	0.077
Septic shock	5.37	2.34–12.31	< 0.001*	2.63	0.53–12.94	0.235
Unknown source of bacteremia	0.44	0.20–0.96	0.04*	8.28	1.86–36.90	0.006*
AKI	2.57	1.08–6.14	0.031*	5.72	1.32–24.88	0.020*
Malignancy	1.21	0.56–2.65	0.628			
APACHE II score ≥ 15	5.55	2.36–13.07	< 0.001*	4.40	0.95–20.38	0.058
AUC_24_ < 420 mg h/l	4.59	1.60–13.18	0.003*	2.09	0.35–12.34	0.417
AUC_ss_ < 420 mg h/l	27.90	7.64–101.85	< 0.001*	44.65	5.59–356.84	< 0.001*

^*^p < 0.05, *AKI* acute kidney injury, *APACHE* acute physiology and chronic health evaluation, *AUC*_*ss*_ area under the curve at steady state, *OR* Odd ratio, *Cl* Confidence interval

## Discussion

This study found that AUCss was the best predicted 30-day mortality by CART analysis. Surprise to the ROC curve of AUC_ss_ is the higher capacity than AUC_ss_/MIC, while the cutoff value in AUC_ss_ and AUC_ss_/MIC is nearly the same. We prefer a cutoff value according to AUC_ss_ (420 mg·h/l). In the real world, MIC is unavailable in all countries, and vancomycin therapeutic drug monitoring in MRSA guideline recommendation keeps AUC/MIC (MIC assumed to be 1 mg/l). When MIC is < 1 mg/l, it is not recommendation decreasing the dose to achieve the AUC/MIC target due to the lack of clinical data and emergence resistance to be reported in AUC < 400 mg﻿·h/l. In addition, when MIC is > 1 mg/l, the probability of achieving an AUC/MIC target of ≥ 400 mg﻿·h/l is low with conventional dosing to prevent nephrotoxicity [10]. Therefore, we recommended keeping AUC for enterococcal infection. We found that AUC_24_ and AUC_ss_ < 420 mg﻿·h/l in patients with AR-VSE bloodstream infection were associated with higher 14-day, 30-day, and in-hospital mortality rates than those with AUC ≥ 420 mg﻿·h/l. In the first 72 h, the outcome of our study was similar to the study by Jumah et al. [11] and Katip et al. [21], who found the 30-day mortality rate in patients with AUC/MIC_Etest_ < 389.08 mg﻿·h/l significantly higher than that of the group of patients with AUC/MIC_Etest_ ≥ 389.09 mg﻿·h/l. In contrast, two other studies did not find a correlation of AUC/MIC with mortality rate [13, 14]. The study by Nakakura et al. [13] found that the Ctr of the surviving group was significantly lower than that of the group that had died at 30 days (14.6 mg/l and 20.5 mg/l, *p* = 0.022). This is consistent with the findings of the study by Sohn et al. [14] that found that Ctr < 15 mg/l was significantly associated with death at 28 days (*p* = 0.041). A retrospective study by Katip et al. includes 300 patients with enterococcal infection and vancomycin administration in Thailand between January 2010 and December 2019. This study showed that a vancomycin trough of < 15 mg/l was associated with significantly higher 30-day mortality (19.28% vs 12.64%, *p* = 0.001) [21]. This is consistent with our study that found that Ctr < 13 mg/l had a tendency for an association with higher mortality at 30 days and significantly higher 14-day mortality rate. The clinical failure rate was not correlated with AUC in our study, which was inconsistent with the findings of Katip et al., who conducted a retrospective study in patients infected with enterococci (bacteremia 11%) receiving vancomycin in Thailand. Of 312 patients, it was found that patients with AUC/MIC ≥ 400 mg﻿·h/l had significantly less clinical failure than those with AUC/MIC < 400 mg﻿·h/l (15.55% and 22.97%, aHR 0.5 [0.26–0.97], *p* = 0.042) [12].

This study found that AUC_ss_ < 420 mg﻿·h/l in a steady state correlated with mortality in patients with enterococcal bacteremia, which is a similar to that for AUC_24_. The 24-h AUC in a steady state had a higher ROC curve than that of 72 h after vancomycin administration, which is good at predicting 30-day mortality. AUC_ss_ < 420 mg﻿·h/l was found to be associated with a 44-fold increase in 30-day mortality rate in multivariate analysis, consistent with a previous study [11]. Our study’s finding is reasonable as the PK/PD target is important for bactericidal activity; low AUC of vancomycin can lead to clinical failure and mortality, probably in association with MRSA infection. Patients with AUC < 420 mg﻿·h/l had a more severe condition than the AUC ≥ 420 mg﻿·h/l group at the first date of vancomycin treatment, which had augmented renal clearance and led to a low serum vancomycin level [10].

This study estimated the AUC of vancomycin using Bayesian software based on the Precise PK platform. The Precise PK calculations incorporated data from previous studies, including Claisse et al. (critically ill patients undergoing renal replacement therapy) [22], Rodvold et al. (patients with varying degrees of renal function) [23], and Westra et al. (patients undergoing hemodialysis) [24]. The patient characteristics in these studies closely aligned with those of the current study population, supporting the validity of the AUC estimation. Bayesian software predicted the AUC from the vancomycin level in the patients’ blood at one point at least, in accordance with the practice guidelines for the therapeutic drug monitoring of vancomycin. Two points (peak-trough) vancomycin concentration was preferred to estimate the Bayesian AUC [10]. A randomized controlled study of 65 subjects by Al-Sulaiti et al. showed that peak-trough concentration had significantly higher therapeutic cure rates than the trough concentration (76.7% vs 48.6%, *p* = 0.02) [25]. Seventy seven patients (68.8%) in our study had two points (peak-trough) of vancomycin concentration for estimating the AUC Bayesian to be accurate and reliable. In contrast, previous studies in enterococcal infection estimated AUC by one point (trough) vancomycin concentration [11–13]. Bayesian dose optimization software uses a well-calculated vancomycin population PK model as the Bayesian prior and the individual patient’s measured drug concentration in the data file to generate a Bayesian posterior parameter value distribution for that patient. PrecisePK and BestDose had the most accurate estimates, but PrecisePK was the least biased [26]. Our study generated AUC using PrecisePK software, but previous studies used BestDose software. This may explain the difference in AUC between the studies [26].

We also found that unknown sources of bacteremia and AKI increase the risk of mortality, consistent with the findings of a study by Uda et al. [15]. Having an unknown source of bacteremia may lead to an inappropriate duration of treatment and failure to properly eliminate the source of infection, which is associated with mortality [2, 3]. A correlation between AKI after vancomycin administration and increased mortality has been reported in patients infected with Gram-positive bacteria [27]. Among 227 patients with vancomycin therapy, 43 cases (19%) developed nephrotoxicity. The AKI patients had higher 28-day mortality than the non-AKI patients (16% vs. 5%, *p* < 0.005) [28]. In the retrospective observational cohort study of patients with MRSA infections who received vancomycin, 40 out of 94 patients developed renal toxicity [29]. The in-hospital mortality rate was found to be significantly higher in nephrotoxicity patients than in those without nephrotoxicity, similar to our study. The causal link between vancomycin-induced AKI and mortality is not clear, but renal dysfunction is a consequence of multiorgan failure, which likely leads to mortality in the critically ill [30, 31].

In this study, the patients with AUC ≥ 700 mg﻿·h/l significantly higher nephrotoxicity rates in the early and later phases. This is consistent with previous studies on enterococci [12] and MRSA infections [10]. It was reported that patients with vancomycin AUC/MIC > 400 mg﻿·h/l in enterococcal infection developed AKI more than three times more often than patients with AUC/MIC ≤ 400 mg﻿·h/l [14.96% vs. 3.85%, aHR 3.96 (1.09–14.47) *p* = 0.037] [12]. Consistent with the study by Katip et al., they found a significantly higher rate of nephrotoxicity in patients with AUC/MIC ≥ 389 mg﻿·h/l or Ctr ≥ 15 mg/l [21]. Among 131 patients with MRSA infection treated with vancomycin in Thailand, the rate of AKI was 19.1%. Vancomycin AUC_ss_/MIC_BMD_ ≥ 698 mg﻿·h/l was found to be associated with a significantly increased risk of AKI, similar to the findings in our study [16]. The data from a previous meta-analysis and updated guidelines for therapeutic monitoring of vancomycin for serious MRSA infection by AUC guide dosing to reducing nephrotoxicity at a value less than 650 [10, 32]. Interestingly, we found that patients with concomitant nephrotoxic drugs have a lower threshold to induce AKI, with a cutoff value of 650 mg﻿·h/l. Vancomycin induced nephrotoxicity by induced apoptosis in porcine proximal tubular cells via mitochondrial production of reactive oxygen species with peroxidation of the mitochondrial phospholipid cardiolipin [33]. This mechanism was dose-dependent, with high AUC related to a high dose to produce more reactive oxygen species. Meanwhile, patients with AKI induced high AUC. Therapeutic drug monitoring is important for patients who receive vancomycin for efficacy and to prevent AKI, which can lead to mortality. We recommended monitoring one point (trough) or 2 points (peak-trough) at the first dose of vancomycin to improve AUC at the early and steady state and suggested that vancomycin AUC in the range of 420–700 mg﻿·h/l is appropriate for efficacy and safety. For patients with concomitant nephrotoxic drugs, we suggest a narrow therapeutic range of AUC (420–650 mg﻿·h/l). Therefore, we recommended a therapeutic range of AUC 420–650 mg﻿·h/l, the same as a recommendation for AUC target for MRSA infection. Because the AUC breakpoint in enterococcal bacteremia is similar to MRSA infection, there is a vancomycin dosing regimen according to practice guidelines for the therapeutic drug monitoring of vancomycin in MRSA infection [10]. Our study was a multicenter study that allowed the inclusion of more participants in enterococcal bloodstream infections than in other studies. Additionally, patients in this study had their vancomycin concentrations monitored at multiple points to calculate the area under the curve (AUC) at both early and steady states, resulting in a more accurate AUC assessment [25]. However, the study has certain limitations. First, this was a retrospective observational study. MIC values were not recorded in all instances, and some data loss and repeated blood cultures were not routinely performed for enterococcal bacteremia. Second, vancomycin assays in this study used two distinct methods from three different settings, which may vary in the results obtained, although vancomycin assays were performed using particle-enhanced turbidimetric inhibitor immunoassay methods in only three cases.

## Conclusion

This study identified the optimal PK/PD target of vancomycin in patients with enterococcal bacteremia as AUC ≥ 420 mg﻿·h/l in the first 72 h and in a steady state. This target correlates with clinical outcomes, such as 14-day, 30-day, and in-hospital mortality rates and microbiological failure. Vancomycin AUC < 650 mg﻿·h/l is recommended to prevent AKI, which is one of the factors associated with increased mortality. Our findings imply that vancomycin levels should be closely monitored and observed clinically to ensure efficacy and safety.

## Data Availability

The datasets used and analyzed during the current study are available from the corresponding author upon reasonable request.

## Abbreviations

PK/PD: Pharmacokinetics/Pharmacodynamics
Ctr: Trough concentration
AKI: Acute kidney injury
Ctr_72h_: Mean trough concentration in the first 72 h
Ctr_ss_: Mean trough concentration in a steady state
AUC/MIC: Area under the curve above minimum inhibitory concentration
AUC_0-24_: AUC in the first 24 h
AUC_24-48_: AUC at 24–48 h
AUC_0-72_: AUC in the first 72 h
AUC_24_: Mean 24-h area under the curve in the first 72 h
AUC_ss_: 24-h area under the curve in a steady state
CART: Classification and Regression Tree
BSI: Bloodstream infection
AR-VSE: Ampicillin-resistant vancomycin-susceptible enterococci
MRSA: Methicillin-resistant *Staphylococcus aureus*
BMD: Broth microdilution
MIC_50_: MIC at the 50th percentile
MIC_90_: MIC at the 90th percentile

## References

[1] Bennett JE, Dolin R, Blaser MJ. Mandell, Douglas, and Bennett's principles and practice of infectious diseases. 9th Edition. Philadelphia: Elsevier; 2019.

[2] Suppli M, Aabenhus R, Harboe ZB, Andersen LP, Tvede M, Jensen JU. Mortality in enterococcal bloodstream infections increases with inappropriate antimicrobial therapy. Clin Microbiol Infect. 2011;17(7):1078–83. 20946408 10.1111/j.1469-0691.2010.03394.x

[3] Cheah AL, Spelman T, Liew D, Peel T, Howden BP, Spelman D, et al. Enterococcal bacteraemia: factors influencing mortality, length of stay and costs of hospitalization. Clin Microbiol Infect. 2013;19(4):E181–9. 23398607 10.1111/1469-0691.12132

[4] Bryan CS, Reynolds KL, Brown JJ. Mortality associated with enterococcal bacteremia. Surg Gynecol Obstet. 1985;160(6):557–61. 4002111

[5] Dubler S, Lenz M, Zimmermann S, Richter DC, Weiss KH, Mehrabi A, et al. Does vancomycin resistance increase mortality in Enterococcus faecium bacteraemia after orthotopic liver transplantation? A retrospective study. Antimicrob Resist Infect Control. 2020;9(1):22. 32005223 10.1186/s13756-020-0683-3 PMC6995054

[6] Bar K, Wisplinghoff H, Wenzel RP, Bearman GM, Edmond MB. Systemic inflammatory response syndrome in adult patients with nosocomial bloodstream infections due to enterococci. BMC Infect Dis. 2006;6:145. 17002792 10.1186/1471-2334-6-145 PMC1592497

[7] López-Luis BA, Sifuentes-Osornio J, Lambraño-Castillo D, Ortiz-Brizuela E, Ramírez-Fontes A, Tovar-Calderón YE, et al. Risk factors and outcomes associated with vancomycin-resistant Enterococcus faecium and ampicillin-resistant Enterococcus faecalis bacteraemia: A 10-year study in a tertiary-care centre in Mexico City. J Glob Antimicrob Resist. 2021;24:198–204. 33359937 10.1016/j.jgar.2020.12.005

[8] Hemapanpairoa J, Changpradub D, Thunyaharn S, Santimaleeworagun W. Does Vancomycin Resistance Increase Mortality? Clinical Outcomes and Predictive Factors for Mortality in Patients with Enterococcus faecium Infections. Antibiotics (Basel). 2021;10(2):105. 33499102 10.3390/antibiotics10020105 PMC7911214

[9] Echeverría-Esnal D, Sorli L, Prim N, Martin-Ontiyuelo C, Horcajada JP, Grau S. Daptomycin versus Glycopeptides for the Treatment of Enterococcus faecium Bacteraemia: A Cohort Study. Antibiotics (Basel). 2021;10(6):716. 34198646 10.3390/antibiotics10060716 PMC8232223

[10] Rybak MJ, Le J, Lodise TP, Levine DP, Bradley JS, Liu C, et al. Therapeutic monitoring of vancomycin for serious methicillin-resistant Staphylococcus aureus infections: A revised consensus guideline and review by the American Society of Health-System Pharmacists, the Infectious Diseases Society of America, the Pediatric Infectious Diseases Society, and the Society of Infectious Diseases Pharmacists. Am J Health Syst Pharm. 2020;77(11):835–64. 32191793 10.1093/ajhp/zxaa036

[11] Jumah MTB, Vasoo S, Menon SR, De PP, Neely M, Teng CB. Pharmacokinetic/Pharmacodynamic Determinants of Vancomycin Efficacy in Enterococcal Bacteremia. Antimicrob Agents Chemother. 2018;62(3):e01602-17. 29263057 10.1128/AAC.01602-17 PMC5826144

[12] Katip W, Oberdorfer P. A Monocentric Retrospective Study of AUC/MIC Ratio of Vancomycin Associated with Clinical Outcomes and Nephrotoxicity in Patients with Enterococcal Infections. Pharmaceutics. 2021;13(9):1378. 34575453 10.3390/pharmaceutics13091378 PMC8464995

[13] Nakakura I, Sakakura K, Imanishi K, Sako R, Yamazaki K. Association between vancomycin pharmacokinetic/pharmacodynamic parameters, patient characteristics, and mortality in patients with bacteremia caused by vancomycin-susceptible Enterococcus faecium: a single-center retrospective study. J Pharmaceutic Health Care Sci. 2019;5(1):8. 10.1186/s40780-019-0138-2 PMC6485087 31093330

[14] Sohn Y, Rim JH, Cho Y, Hyun J, Baek Y, Kim M, et al. Association of vancomycin trough concentration on the treatment outcome of patients with bacteremia caused by Enterococcus species. BMC Infect Dis. 2021;21(1):1099. 34702193 10.1186/s12879-021-06809-x PMC8547083

[15] Uda A, Shigemura K, Kitagawa K, Osawa K, Onuma K, Yan Y, et al. Risk Factors for the Acquisition of Enterococcus faecium Infection and Mortality in Patients with Enterococcal Bacteremia: A 5-Year Retrospective Analysis in a Tertiary Care University Hospital. Antibiotics (Basel). 2021;10(1):64. 33440660 10.3390/antibiotics10010064 PMC7826794

[16] Chattaweelarp T, Changpradub D, Punyawudho B, Thunyaharn S, Santimaleeworagun W. Is Early Monitoring Better? Impact of Early Vancomycin Exposure on Treatment Outcomes and Nephrotoxicity in Patients with Methicillin-Resistant Staphylococcus aureus Infections. Antibiotics (Basel). 2020;9(10):672. 33020463 10.3390/antibiotics9100672 PMC7601693

[17] Chavada R, Ghosh N, Sandaradura I, Maley M, Van Hal SJ. Establishment of an AUC(0–24) Threshold for Nephrotoxicity Is a Step towards Individualized Vancomycin Dosing for Methicillin-Resistant Staphylococcus aureus Bacteremia. Antimicrob Agents Chemother. 2017;61(5):e02535-16. 28242672 10.1128/AAC.02535-16 PMC5404579

[18] Khwaja A. KDIGO clinical practice guidelines for acute kidney injury. Nephron Clin Pract. 2012;120(4):c179–84. 22890468 10.1159/000339789

[19] Lodise TP, Drusano GL, Zasowski E, Dihmess A, Lazariu V, Cosler L, et al. Vancomycin exposure in patients with methicillin-resistant Staphylococcus aureus bloodstream infections: how much is enough? Clin Infect Dis. 2014;59(5):666–75. 24867791 10.1093/cid/ciu398

[20] CLSI. Performance Standards for Antimicrobial Susceptibility Testing. 33rd ed. CLSI supplement M100. Clinical and Laboratory Standards Institute. 2023.

[21] Katip W, Okonogi S, Oberdorfer P. The Thirty-Day Mortality Rate and Nephrotoxicity Associated With Trough Serum Vancomycin Concentrations During Treatment of Enterococcal Infections: A Propensity Score Matching Analysis. Front Pharmacol. 2021;12:773994. 35153743 10.3389/fphar.2021.773994 PMC8831381

[22] Claisse G, Zufferey PJ, Trone JC, Maillard N, Delavenne X, Laporte S, et al. Predicting the dose of vancomycin in ICU patients receiving different types of RRT therapy: a model-based meta-analytic approach. Br J Clin Pharmacol. 2019;85(6):1215–26. 30768726 10.1111/bcp.13904 PMC6533443

[23] Rodvold KA, Blum RA, Fischer JH, Zokufa HZ, Rotschafer JC, Crossley KB, et al. Vancomycin pharmacokinetics in patients with various degrees of renal function. Antimicrob Agents Chemother. 1988;32(6):848–52. 3415206 10.1128/aac.32.6.848 PMC172294

[24] Westra N, Proost JH, Franssen CFM, Wilms EB, van Buren M, Touw DJ. Vancomycin pharmacokinetic model development in patients on intermittent online hemodiafiltration. PLoS ONE. 2019;14(5):e0216801. 31086400 10.1371/journal.pone.0216801 PMC6516654

[25] Al-Sulaiti FK, Nader AM, Saad MO, Shaukat A, Parakadavathu R, Elzubair A, et al. Clinical and Pharmacokinetic Outcomes of Peak-Trough-Based Versus Trough-Based Vancomycin Therapeutic Drug Monitoring Approaches: A Pragmatic Randomized Controlled Trial. Eur J Drug Metab Pharmacokinet. 2019;44(5):639–52. 30919233 10.1007/s13318-019-00551-1 PMC6746691

[26] Turner RB, Kojiro K, Shephard EA, Won R, Chang E, Chan D, et al. Review and Validation of Bayesian Dose-Optimizing Software and Equations for Calculation of the Vancomycin Area Under the Curve in Critically Ill Patients. Pharmacotherapy. 2018;38(12):1174–83. 30362592 10.1002/phar.2191

[27] Jeffres MN. The Whole Price of Vancomycin: Toxicities, Troughs, and Time. Drugs. 2017;77(11):1143–54. 28573434 10.1007/s40265-017-0764-7 PMC5501899

[28] Minejima E, Choi J, Beringer P, Lou M, Tse E, Wong-Beringer A. Applying new diagnostic criteria for acute kidney injury to facilitate early identification of nephrotoxicity in vancomycin-treated patients. Antimicrob Agents Chemother. 2011;55(7):3278–83. 21576448 10.1128/AAC.00173-11 PMC3122454

[29] Jeffres MN, Isakow W, Doherty JA, Micek ST, Kollef MH. A retrospective analysis of possible renal toxicity associated with vancomycin in patients with health care-associated methicillin-resistant Staphylococcus aureus pneumonia. Clin Ther. 2007;29(6):1107–15. 17692725 10.1016/j.clinthera.2007.06.014

[30] Zhang Y, Du M, Chang Y, Chen L-a, Zhang Q. Incidence, clinical characteristics, and outcomes of nosocomial Enterococcus spp. bloodstream infections in a tertiary-care hospital in Beijing, China: a four-year retrospective study. Antimicrob Resist Infect Control. 2017;6(1):73. 10.1186/s13756-017-0231-y PMC5496248 28680588

[31] Uchino S, Kellum JA, Bellomo R, Doig GS, Morimatsu H, Morgera S, et al. Acute Renal Failure in Critically Ill PatientsA Multinational. Multicenter Study JAMA. 2005;294(7):813–8. 16106006 10.1001/jama.294.7.813

[32] Aljefri DM, Avedissian SN, Rhodes NJ, Postelnick MJ, Nguyen K, Scheetz MH. Vancomycin Area Under the Curve and Acute Kidney Injury: A Meta-analysis. Clin Infect Dis. 2019;69(11):1881–7. 30715208 10.1093/cid/ciz051 PMC6853683

[33] Filippone EJ, Kraft WK, Farber JL. The Nephrotoxicity of Vancomycin. Clin Pharmacol Ther. 2017;102(3):459–69. 28474732 10.1002/cpt.726 PMC5579760

